# Erratum

**DOI:** 10.1590/0104-1169.0000.2501

**Published:** 2014

**Table:** 

Issue	Page	For	Read
v21nSpec	143	Few studies address preterm infants’ breast feeding performance^(14-15)^. In the present study, we considered as the “gold standard” when the infant was able to feed 5ml of breast milk. This decision was based on previous studies that considered efficient sucking at the maternal breast as either milk intake of 5ml with the presence of sucking movements upon the preterm’s initial contact with the mother^(16)^, or the presence of oral milk intake, independently of the device used (bottle, cup or bottle with fine nipple)^(7)^.	Few studies address preterm infants’ breast feeding performance^(14-16)^. In the present study, we considered as the “gold standard” when the infant was able to feed 5ml of breast milk. This decision was based on previous studies that considered efficient sucking at the maternal breast as either milk intake of 5ml with the presence of sucking movements upon the preterm’s initial contact with the mother, or the presence of oral milk intake, independently of the device used (bottle, cup or bottle with fine nipple)^(7)^.
	144	Studies have used the weight difference before and after breast feeding to verify the milk volume ingested by the preterm^(3,16-17)^.	Studies have used the weight difference before and after breast feeding to verify the milk volume ingested by the preterm^(3,17)^.

